# Suture fixation of tracheal stents for the treatment of upper trachea stenosis: a retrospective study

**DOI:** 10.1186/s13019-018-0790-x

**Published:** 2018-11-09

**Authors:** Jingtao Huang, Zhongwei Zhang, Tao Zhang

**Affiliations:** Department of Thoracic Surgery, Tianjin Nankai Hospital, No. 6 Changjiang Road, Nankai District, Tianjin, 300100 China

**Keywords:** Tracheal stenosis, Tracheal stent, Stent migration, Suture fixation, Complication

## Abstract

**Background:**

Stent migration is a common complication in treating trachea stenosis. There is no report concerning suture fixation of tracheal stent. The aim of this study was to investigate whether suture fixation of tracheal stent could avoid stent migration in patients with upper trachea stenosis. The complications were further investigated.

**Methods:**

The patients with upper trachea stenosis who underwent tracheal stent placement for benign/malignant conditions in our hospital between May 2016 and April 2018 were retrospectively reviewed. Clinical data were collected for each patient, including age, gender, co-morbid diseases, site of tracheal obstruction, degree of tracheal obstruction, success of stent placement, impact on patient’s symptoms, complications, etc.

**Results:**

Eleven patients (8 males and 3 females; range of age: 17–85, and average age of 63) were enrolled into this study. Six silicone stents and five membrane-covered metal stents were used. The surgery was successfully performed in all the cases. The postoperative recovery was uneventful. All symptoms of the patients were relieved. No complications occurred. The average follow-up for patients was 5 months (range of 1–13 months). During the follow-up, no stent migration was observed according to CT and bronchoscope.

**Conclusion:**

The results suggested that suture fixation of stents could avoid stent migration in treating upper trachea stenosis with metal stent or silicone stent. This method seemed to be effective without operation complications.

## Background

Tracheal stenosis refers to abnormal narrowing of the central air passageways, [1] which can occur at the larynx, trachea, carina or main bronchi [2]. The causes for tracheal stenosis are various and the optimal therapy is not well defined, depending largely on the type of the stenosis [3]. Generally, in some very experienced surgery centers, tracheal resection and reconstruction might be considered as the best treatment to completely cure the stenosis and allows to obtain good results [4]. For example, it is available in tracheal stenosis resulting from prolonged endotracheal intubation or tracheostomy [5]. Nowadays tracheal stenting is a preferred alternative, which can be used for patients with malignant stenosis and benign stenosis [6]. Airway stents are the most common tracheobronchial prosthesis and can be inserted into the trachea. They can rapidly relieve the narrowing air passageway and breathing difficulty [7, 8].

With improved design of stents and advanced technology to aid in the stent insertion, more patients are being treated with tracheal stents. However, numerous complications associated with the use of stents have been reported, including partial or complete trachea obstruction, stent migration and infections [9]. Among those complications, migration of the stent is very common [10]. Martinez-Ballarin et al. reported the rate of migration of stents as 17.5%, [11] and Park et al. found migration of stents in 34% patients [9]. Migration of silicone stents is reported to be 16.6–24%, [12, 13] and migration of metal stent is reported as 2.2–6.4% [12, 14]. Stent migration brings a lot of pain to the patient and increase therapy cost. Silicone Y-stents can be used to inhibit the stent migration, however they are only reserved for the patients with stenosis around the area of tracheal carina [7]. Therefore, it is a critical issue to avoid tracheal stent migration.

Although there are many reports on the efficacy and complications of stents for the treatment of trachea stenosis, as far as we know, there is no report concerning suture fixation of tracheal stent in treating upper trachea stenosis. Since May 2016, our institution has been using suture fixation of stent to treat patient with upper trachea stenosis. Our clinical experience suggested that it seemed to be an effective method. Therefore, we retrospectively reviewed the patients who underwent suture fixation of tracheal stent in order to see if it could avoid stent migration. The complications were also investigated.

## Methods

### Patients

This study was approved by the ethic committee of our hospital. The patients with upper trachea stenosis who underwent tracheal stent placement for benign/malignant conditions in our hospital between May 2016 and April 2018 were retrospectively reviewed. The inclusion criteria were patients with upper trachea stenosis and patient who underwent suture fixation of the stent.

As for diagnosis of trachea stenosis, it was evaluated with computed tomography and bronchoscopy. According to CT images, the cross section area of the narrowest part of the trachea (ANWT) and the cross section area of the normal part of the trachea (ANT) were evaluated. The stenosis degree was defined as ANWT/ANT. Each patient underwent a standard assessment, including physical examination, routine laboratory tests, chest radiography and chest computed tomography prior to the stenting operation. In our institute, the indications for stent placement were trachea stenosis > 50% and dyspnea above grade III. The grade of dyspnea was assessed in reference to World Health Organization (WHO). The indications for suture fixation in this study were patients with upper tracheal stent migration and the patient who needs upper tracheal stenting.

### Surgery procedures

**Fig. 1:**
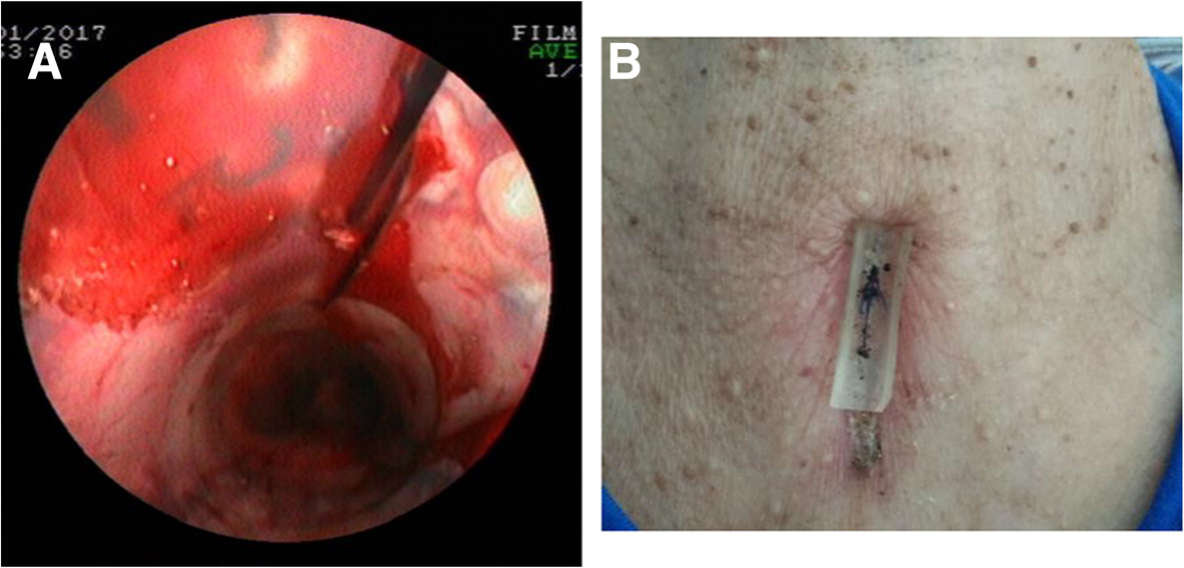
Images of the operation procedures. **a**: Suture fixation method for the tracheal stent. **b**: Postoperative CT showing the silicon pad on the skin at the puncture site (as the arrow indicates)

After general anesthesia, the patients were placed in trendelenburg position. A rigid bronchoscope was inserted into the trachea under the guidance of a flexible bronchoscope. Ventilation was assured by using a ventilator. After tracheal stenosis was treated with cryoresection, acusection, argon plasma coagulation, balloon dilatation properly, the membrane-covered metal stents (Micro-Tech Co. Ltd., Nanjing, China) or Dumon stent (Novatech, La Ciotat, France) were placed during a rigid bronchoscopy procedure. Under endoscope, a double-needle gastropexy device of PEG kit (Create Medic Co., Ltd., Yokohama, Japan) was applied. The puncture was performed at the part between the inferior margin of the cricoid cartilage and the suprasternal notch. After the position of stent was adjusted satisfactory, the anterior of the stent was fixed onto the antetheca of the trachea and its front tissue under endoscopic monitoring. To prevent surgical suture from cutting the skin, a silicon pad (width of 1–1.5 cm and length of 3-4 cm) was placed on the skin at the puncture site (Fig. 1). A suture fixation about 1–3 stitches were done. Postoperatively, phone call following-up was done every month to see if there’s any complication.

In the patients with benign stenosis, the suture was removed under the following conditions. Silicon stent was inserted for more than 3 months; the uncovered part of metal stent was covered by granulation scar; the patient asked for suture removal.

### Data collection

Demographic and clinical data were collected for each patient, including age, gender, co-morbid diseases, site of trachea obstruction, degree of trachea obstruction, previous interventional bronchoscopic procedures (including or not including trachea stenting), duration of procedure, success of stent placement, impact on patient’s symptoms, procedure- and stent-related complications, duration of follow-up, and the final outcomes.

## Results

### Basic information of the patients

Eleven patients (8 males and 3 females; range of age: 17–85, and average age of 63) met the inclusion criteria and were enrolled into this study. The upper margins of the stenosis were at 2–5 cm below the glottis. The main symptoms of those patients were dyspnea of grade V. Stent migration occurred in two patients at 1 day and 46 days after the first stent implantation respectively. Those two patients were treated with stent implantation again and suture fixation was performed. The characteristics of the patients were listed in Table 1.

**Table 1:** General information of the patients

Case No.	Age	Gender	B/M	Medical History (month)	Location of the stenosis/degree of stenosis (90%)	Length of the stenosis (mm)	Previous interventional procedures
1	85	F	M	10	Middle&Upper/95	30	Yes
2	74	M	B	3	Upper/90	20	No
3	84	F	M	3	Middle&upper/90	20	No
4	75	M	B	11	Upper/90	25	Yes
5	28	F	B	6	Upper/90	20	no
6	54	M	M	2	Upper/95	40	No
7	81	M	M	1	Upper/90	40	No
8	17	M	B	2	Upper/95	50	No
9	75	M	M	3	Upper/90	30	No
10	66	M	M	2	Middle&upper/95	20	No
11	55	M	M	6	Upper/95	30	No

*F* female, *M* male, *B* benign, *M* malignant

### Surgery profile and results

The surgery was successfully performed in the 11 cases. The suture fixation of the stent took about 5-15 min. During the operation, oxyhemoglobin saturation was stable and no haemorrhage was observed. The patients were managed in the post-anesthesia care unit and they were then sent to the ward after the vital parameters were normal. The postoperative recovery was uneventful, and the patient was discharged from hospital after 4–18 days (average: 8 days) of observation (Table 2). The patients who only had treatment for tracheal stenosis were discharged within 5 days postoperatively; those having other treatments had longer hospital stay. Post operatively, the patients’ symptoms were relieved.

**Table 2:** Intraoperative and postoperative information of the patients

Case No.	Stenosis treatment	Stent Type/stent diameter (mm)/ stent length (mm)	VAS	Suture removal after the stent replacement (month)	Postoperative hospital stay (day)	Postoperative survival time (month)	Follow-up (month)
1	Cryoresection, APC	Silicone/16/50	2	/	5	5	5
2	Acusection, balloon dilatation	Silicone/16/50	3	4	4	/	8
3	Cryoresection, APC	MCMS /18/40	1	/	17	7	7
4	Acusection, balloon dilatation	Silicone/16/50	2	6	5	/	6
5	Acusection, balloon dilatation	Silicone/16/50	1	12	12	/	13
6	Cryoresection, APC	MCMS /20/60	1	/	14	6	6
7	Balloon dilatation	MCMS /20/60	1	/	4	4	4
8	Acusection, balloon dilatation	Silicone/16/60	2	/	5	/	3
9	/	MCMS /20/60	2	/	18	1	1
10	Balloon dilatation	MCMS /18/60	1	/	5	Under radiotherapy	1
11	Balloon dilatation	Silicone/16/60	3	/	4	Under chemotherapy	1

*VAS* Visual Analogue Scale, *MCMS* membrane-covered metal stents, *APC* argon plasma coagulation

The suture was successfully removed in the patients with benign stenosis at 4 months, 6 months and 12 months after stent placement respectively. The stents didn’t migrate after the suture removal.

### Complications

Postoperatively, there was slight hyperemia around the silicon pad due to the pressure from the suture in four patients. Regarding this, the patients had mild discomfortable feeling in the suture site, which didn’t affect coughing or deglutition. The discomfortableness was relieved within 3–5 days. As for pain evaluation, Visual Analogue Scale (VAS) was used; the results were detailed in Table 2. All patients were satisfactory with the results. No other complications were observed.

### Follow-up results

**Fig. 2:**
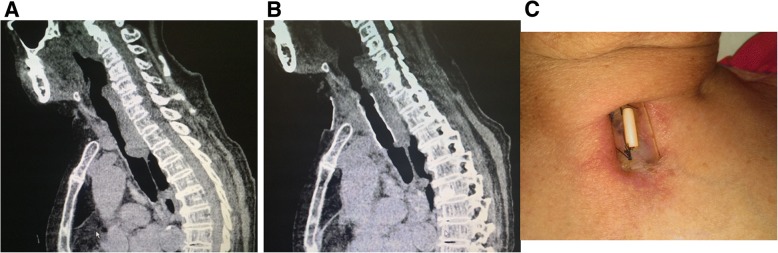
Preoperative and postoperative images of one patient (Case No. 1). **a**: Preoperative sagittal CT showing the upper tracheal stenosis. **b**: Postoperative sagittal CT showing the normal trachea. **c**: The appearance of the suture fixation on the neck

**Fig. 3:**
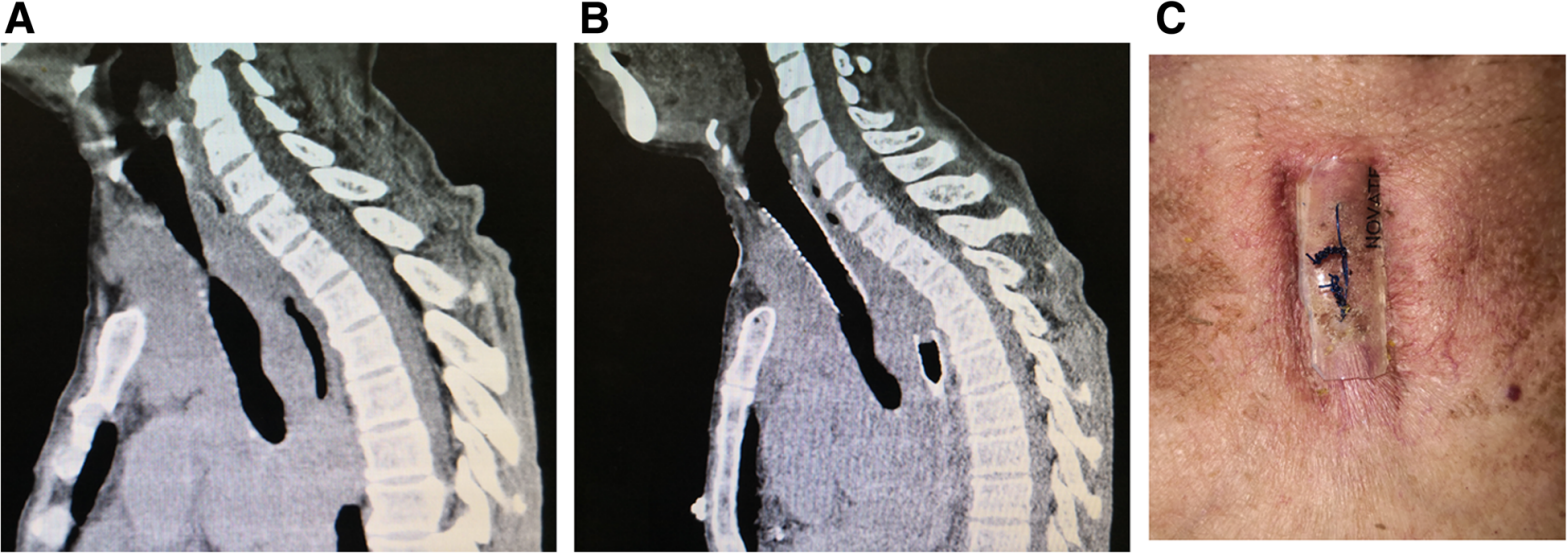
Preoperative and postoperative images of one patient (Case No. 9). **a**: Sagittal preoperative CT showing the upper tracheal stenosis. **b**: Postoperative sagittal CT showing the normal trachea. **c**: The appearance of the suture on the neck

The average follow-up for patients was 5 months (range of 1–13 months). During the follow-up, no stent migration was observed according to CT and bronchoscope (Figs. 2 and 3). The patients’ symptoms disappeared.

## Discussion

Migration of stent is still a serious clinical issue, with a reported migration rate of 16.6–24% in silicone stents [12, 13] and 2.2–6.4% in metal stent [12, 14]. Thus, it calls for effective measures to prevent the tracheal stents from migrating. In this study, we mainly found that suture fixation of stents avoided stent migration in patients with upper trachea stenosis. The patients’ symptoms were successfully relieved without stent migration.

To prevent tracheal stent from migrating, the studs are particularly designed in the silicon stent. Still it can’t completely avoid stent migration. In fact, there are several factors that may contribute to migration of stent. Firstly, we found that the middle and lower trachea appeared wider than upper trachea according to CT 3D reconstructions in those patients, which might be attributed to the migration of the upper tracheal stent. Besides, with the rather narrow lumen in the stenosis site, the stenosed segment may exert more compression to the stent. Secondly, patients with tracheal disease usually suffer cough which may contribute to stent migrating. Thirdly, the gravity of the stent is also a contributing factor. Fourthly, another cause is the higher pressure in the superior of the glottis than in the inferior part. In the past, we would use a longer stent or a stent with larger-diameter when there’s stent migration, which however might still failed to avoiding stent migration. Besides, a longer stent might result in sputum drainage disorder in some patients and then the stent had to be removed. It brought a lot of pain and risk to the patients.

In fact, there are some researches concerning the methods for external fixation of tracheal stents [15–18]. For example, Colt et al. described a technique of external fixation of subglottic stents, which appeared to be applicable to some carefully-selected patients with subglottic stenosis who have failed indwelling stent placement because of stent migration [15]. Miwa et al. reported a method utilizing an external fixation apparatus for silicone stents in the subglottic trachea [17]. Majid et al. suggested a technique of securing endoluminal stents using an Endo Close suturing device (Coviden, Boston, MA) and an external silicone button. And their method was proved to be effective in nine patients [16]. Considering the efficacy and surgical complications, we have been using suture fixation of the stent in recent years. According to our clinical experience, it seemed that the present method was effective and could avoid stent migration without any complication. There are some points requiring careful attention when perform this method. To gain a better suture fixation, the acupuncture should be perpendicular to the course of the upper trachea. Besides, the patients were placed in trendelenburg position when implanting the stent, in which condition the neck skin was tracked slightly to the head. Therefore, the shoulder pad should be removed before acupuncture to guarantee satisfactory suture fixation.

The suture in the neck would produce certain tension with patients’ swallowing and coughing. Besides, the suture would have a long retention in the neck. Thus, it cut the neck skin easily and may even cut into the subcutaneous part. To reduce the stress of the suture on the neck skin, a silicon pad was place on the skin at the fixation site in this study. Postoperatively, there was only slight hyperemia around the silicon pad in four patients. Although it gave the patients some mild discomfortable feelings in the suture part, it didn’t affect normal function of coughing or deglutition. The discomfortableness was relieved within 3–5 days. All patients were satisfactory with the results. As for removing the suture, it was done in the patient with benign stenosis in this study. On the contrast, in the patients with malignant stenosis, it’s difficult to well fix the stent without the suture because of the softness of the malignant tumor. Besides, considering the short lifetime of those patients, the suture were not removed in the present patients with malignant stenosis. As for the patients with benign stenosis, silicone stents were used in the present study. After the suture fixation for a proper period, the trachea and stent could remain stable even if the suture fixation was removed. In this study, the suture was removed at 4 months, 6 months and 12 months after stent placement respectively. The stents didn’t migrate after the suture removal. However, it’s uncertain when to remove the sure. It’s far from drawing a solid conclusion due to the very small sample size. According to our clinical experience, we suggested the following three criteria. Firstly, silicon stent was inserted for more than 3 months; the uncovered part of metal stent was covered by granulation scar; the patient asked for suture removal. It still needs close attention in further clinical practice to determine the proper criteria for suture removal.

As for complications following the stent placement, Martinez-Ballarin et al. found migration of stents in 17.5%, granulation tissue formation in 6.3%, and trachea obstruction due to mucostasis in 6.3% of cases [11]. Park et al. found restenosis in 40%, granulation tissue formation in 38%, migration of stents in 34%, and mucostasis in 31% of cases [9]. The formation of tracheoesophageal fistula (TEF) has been reported after placement of esophageal stent for stricture esophagus and metallic tracheal stent for tracheal stenosis [19]. The probable cause of fistula formation may be due to the injury at the time of stent deployment or erosion of the tracheal/bronchial wall by proximal/distal margin of a malpositioned stent for prolonged time. In present study, no complications occurred except for slight hyperemia around the silicon pad which was relieved within 3–5 days.

Besides, this suture fixation procedure is relatively easy to perform and usually take short operation time, that is several minutes. Thus, during our clinical experience, this method was used to the patients with upper tracheal stent migration and the patient who needs upper tracheal stenting, in order to avoid stent migration. There are several limitations in this study. Except for the nature of retrospective study, the sample size in this study was too small to draw a highly valid conclusion. The follow-up is not very long in some cases at present; those patients are still under our observation. A further study with a larger sample size is needed to further verify the efficacy of this method.

## Conclusion

The results suggested that suture fixation of stents could avoid stent migration in treating upper trachea stenosis with metal stent and silicone stent. This method seemed to be effective without operation complications.

## Abbreviations

ANT: The cross section area of the normal part of the trachea
ANWT: The cross section area of the narrowest part of the trachea
APC: Argon plasma coagulation
MCMS: Membrane-covered metal stents
VAS: Visual Analogue Scale
WHO: World Health Organization
